# Association between cardiovascular risk factors and cognitive impairment in adults aged 60 years or older from Chile: a cross-sectional study

**DOI:** 10.1186/s12877-023-04410-2

**Published:** 2023-12-05

**Authors:** Josivaldo Souza-Lima, Sandra Mahecha Matsudo, Pedro Valdivia-Moral, Waldo Pérez, Clemens Drenowatz, Jorge Sapunar Zenteno, Gerson Ferrari

**Affiliations:** 1 Facultad de Educación, Universidad de Granada, Granada, 18071 España; 2 Facultad de Ciencias, Universidad Mayor, Santiago, Chile; 3 Hémera Centro de Observación de la tierra, Facultad de Ciencias Universidad Mayor, Santiago, Chile; 4 Division of Sport, Physical Activity and Health, University of Education Upper Austria, Linz, Austria; 5 Centro de Excelencia de Medicina Translacional, Facultad de Medicina, Universidad de La Frontera, Chile; 6 Facultad de Ciencias de la Salud, Universidad Autónoma de Chile, Providencia, Chile

**Keywords:** Epidemiology, Latin America, objective cognitive function, Cardiovascular risk factors, Elderly, Public health

## Abstract

**Background:**

Few studies in Latin America have examined the association between cardiovascular risk factors and cognitive impairment (CI) in a nationally representative sample. Therefore, this study aimed to estimate the prevalence of CI in a nationally representative sample of adults aged 60 years or older from Chile and to investigate the association between cardiovascular risk factors and CI.

**Methods:**

Data from the cross-sectional 2016–2017 National Health Survey of Chile, which included 2031 adults (63.7% women) was used. Body mass index, metabolic syndrome (blood pressure, triglycerides, fasting glucose or treatment for diabetics, waist circumference, and HDL cholesterol), risk of cardiovascular disease (history and measured variables, using the Framingham risk score), tobacco use, and physical activity were measured. CI was assessed using the Mini-Mental Status Examination (MMSE).

**Results:**

Overall, the prevalence of CI was 12.2% at the national level. Significant differences in CI were observed by age, education level, risk of cardiovascular disease, and smoking. High risk of cardiovascular disease was associated with higher odds of CI (OR: 2.04; 95%CI: 1.20–3.45) compared to low risk. Smoking was significantly associated with a lower likelihood of CI (OR: 0.56; 95%CI: 0.36–0.87) compared to never smoking. Body mass index, metabolic syndrome, and physical activity were not associated with CI.

**Conclusions:**

This study provided additional support for previous findings on the relationship between cognitive decline and an elevated risk of cardiovascular disease. Worse CI was associated with the group with the highest risk of cardiovascular disease, and the presence of lifestyle factors, such as obesity and physical inactivity, exacerbate this relationship, but not being a current smoker.

## Background

The prevalence of cognitive impairment (CI), which may lead to dementia including Alzheimer’s, vascular dementia, and others, has been increasing worldwide [1, 2]. Objective cognitive function impairment refers to the decline in mental abilities, including memory, attention, perception, and reasoning, which may occur with age or due to certain health conditions [3].

CI is currently the seventh leading cause of death among all diseases and one of the major causes of disability and dependency among older people worldwide [4]. CI has severe consequences for individuals, their families, the health care system, and the economy [4]. CI is commonly diagnosed in elderly people, and it is estimated that by 2050 there will be more than 100 million people diagnosed with CI [5]. Given its high global prevalence, early diagnosis could save the public health system an estimated 7 trillion dollars in spending on long-term medical care [6].

Previous studies suggests that CI is affected by several factors, such as neurodegenerative diseases, cardiovascular and cerebrovascular diseases, infections, trauma, and tumors [7]. However, there are other important factors in the pathogenesis of dementia such as a sedentary lifestyle, lack physical activity, poor nutrition and social or environmental factors [8]. For instance, it has been calculated that about 3% of all dementia cases could be prevented by increasing levels of physical activity [9, 10].

Estimates suggest that in the Latin American region the prevalence of dementia is around 7%, affecting more women than men (7.7% vs. 5.9%) [11]. Particularly in Chile, the mortality rate linked to dementia has increased fivefold in 20 years [12]. The country has experienced a notable increase in life expectancy, resulting in a growing aging population facing an increased burden of non-communicable diseases, particularly among the elderly [13].

Chile has experienced considerable changes in people’s lifestyle and lifespan, urbanization, and environment, which are likely to affect exposure to risk factors for CI and thus the prevalence of CI [14–16]. For example, a higher prevalence of physical inactivity and obesity among Chilean individuals in recent years has led to increases in cardiovascular risk factors and several diseases, which are associated with CI [17]. Previous studies, however, have not assessed the association between cardiovascular risk factors and CI, particularly in low- and middle-income countries. Additionally, few reports have focused on the management of CI, making this a poorly understood, yet crucially important, topic. Compelling evidence has accumulated on the critical role of healthy lifestyle factors for the maintenance of cognitive health and in the prevention of cognitive decline as well as the progression of CI [18].

Epidemiologic studies can provide a better knowledge of the risk variables linked with cognitive decline in older adults, which is critical given the expanding elderly population. About 80% of the Indigenous population lives in urban areas and practically 50% live in the Metropolitan region, which is the most populated in the country such studies may have important implications for developing preventative and therapeutic programs for cognitive health in urban environments [19]. On the other hand, national level or large geographical area estimates do not account for the disease variability in smaller geographical areas within a country, potentially missing information and target populations. At the Latin American level, few studies focus solely on a Latin American population, emphasizing its regional significance and can help to understand the special issues that Latin American communities confront in terms of cognitive health, and they can be compared to other studies undertaken in different nations around the region [20].

In this study, we aimed to estimate the prevalence of CI in a large, nationally representative sample of older adults in Chile. We also sought to examine the association of cardiovascular risk factors with objective cognitive function, considering variables such as sex, age, educational level, marital status, and ethnicity. By analyzing these sociodemographic factors alongside cardiovascular risk factors, we aim to provide a comprehensive understanding of the prevalence and potential risk factors associated with CI in the Chilean older adult population.

## Methods

### Study design and sample

This cross-sectional study uses data from participants from the 2016–2017 Chilean National Health Survey (CNHS). The CNHS is a nationally representative population-based household survey on health behaviors [11]. A complex, multistage sampling strategy was performed, considering counties as the primary sampling unit, district and households as the secondary and thirdly, one participant from selected households as sampling unit. Sampling weights from the survey accounted for differences in selection probability and non-response rates. The post-stratification adjustment allowed to expand the sample to the estimated population in Chile. Chile is geographically divided into 16 regions: Arica y Parinacota, Tarapacá, Antofagasta, Atacama, Coquimbo, Valparaíso, Metropolitana de Santiago, Libertador General Bernardo O´Higgins, Maule, Bio Bio, Araucania, Los Rios, Los Lagos, Aysén and Magallanes. The CNHS did not include the Ñuble region. Data collection was carried out between August 2016 and March 2017. One participant per household was randomly selected using a Kish computational algorithm.

The study protocol was approved by the Research Ethics Committee of the Faculty of Medicine of Pontificia Universidad Católica de Chile (project number 16–019), and written informed consent was obtained before data collection. All aspects of the study were conducted in accordance with the Declaration of Helsinki. We did a secondary analysis study based on the CNHS dataset through the Transparency Law, which enables data availability via a formal request at http://epi.minsal.cl/bases-de-datos/. Details about CNHS are available elsewhere [11].

**Fig. 1:**
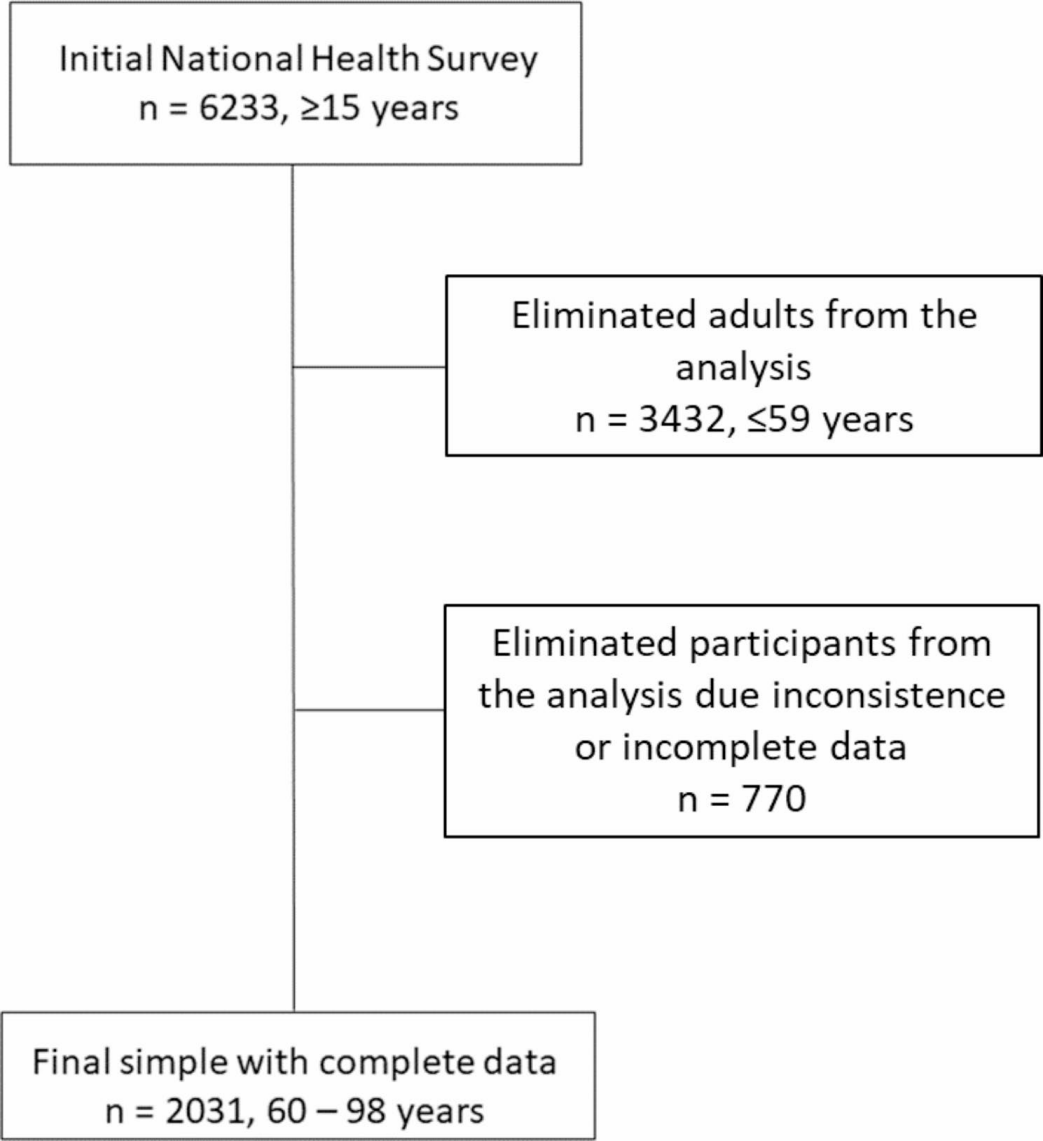
Flow chart of the process to obtain the final sample from the National Health Survey

The CNHS included 6233 participants aged ≥ 15 years and used a stratified and multistage selection of participants. In our study, we eliminated participants between 15 and 59 years of age (n = 4202) due to: (1) difference of prevalence of objective cognitive function compared with people aged ≥ 60 years; (2) CI being a major public health concern in the elderly population; (3) I being a major cause of disability, dependency, and poor quality of life [21]. Participants with missing data on cardiovascular risk factors and CI were eliminated (n = 770). Thus, the final sample for the statistical analyses included 2031 participants between 60 and 98 years of age (Fig. 1).

### Cardiovascular risk factors

Cardiovascular risk factors included in this study were body mass index, metabolic syndrome (including waist circumference, low HDL, hypertension, and impaired fasting glucose), risk of cardiovascular disease based on the Framingham criteria, tobacco use, and physical activity.

Body mass index was obtained by using objective measures of height and body weight. A medical device that is specifically designed to measure a person’s height accurately (portable stadiometer) was used to measure the height (closest to 0.1 centimeters) [11]. Weight was measured using a digital scale (OMRON HN 289) with a precision of 0.1 kg. Measurements were taken with the participants barefoot and wearing light clothing. Body mass index was calculated as weight/height^2^ and participants were classified as underweight (< 18.5 kg/m^2^), normal weight (18.5–24.9 kg/m^2^), overweight (25.0-29.9 kg/m^2^), and obese (≥ 30.0 kg/m^2^) [11].

Metabolic syndrome was defined according to the Chilean National Guidelines as having at least three of the following five components: high waist circumference (> 90 cm for men and > 80 cm for women), low HDL (< 40 mg/dl in men and < 50 mg/ dl in women), hypertension (130/85 mmHG or under BP-lowering treatment) and impaired fasting glucose (IFG, glucose > 5.6 mmol/L or under treatment with antidiabetic drugs) [11]. The classification criteria for metabolic syndrome used in this study include the presence of three or more of the following conditions: abdominal obesity, high blood pressure, high blood sugar, high triglyceride levels, and low levels of high-density lipoprotein cholesterol (HDL).

Cardiovascular disease risk of the participants was calculated using the Framingham criteria that include age, sex, use of tobacco, total cholesterol, HDL cholesterol, systolic blood pressure and use of pharmacological treatment to control high blood pressure. participants were classified into low risk (< 10%), moderate risk (10–20%), or high risk (> 20%). A Framingham risk score of ≤ 4 points was considered low risk, between 5 and 9 points was considered moderate risk and ≥ 10 points was considered high risk. The method was validated in the Latin American population and used in other studies [11, 22–25].

Smoking was derived from the following question: “Do you currently smoke Cigarettes?” (Occasionally, < 1 Cigarette a day, > 1 Cigarette a day, quit smoking, never smoked). Responses were subsequently classified as never smoking, ex-smoker or smoker) [11].

The Global Physical Activity Questionnaire (GPAQ) was used to quantify physical activity. Specifically, participants reported duration, frequency, and intensity of activities in the domains occupational PA, active commuting, and recreational PA. For each domain the metabolic equivalent of tasks (METs; where 1 MET = ~ 3.5 ml O^2^ kg^− 1^ Min^− 1^) were assigned based on the questionnaire protocol (3.3-MET was used for light activities, 4.0-METs was used for moderate activities, 6.0-METs was used for cycling-related activities, and 8.0-METs for vigorous activities). Total self-reported physical activity was calculated as the sum of MET-min/week^− 1^ across the three domains, and participants were subsequently classified as physically inactive (< 600 MET-min/week^− 1^) or active (≥ 600 MET-min/week^− 1^) [26, 27].

### Cognitive impairment

The recognition and assessment of people with suspected CI requires a test of cognitive function or the use of informant questionnaires, or both [28, 29]. Brief cognitive evaluations resulting in an objective score are easy to administer, take no longer than 10 min to complete and address major executive functions. Such a final score is useful in determining which individuals need a more comprehensive evaluation [30]. One of these brief CI tests is the Mini-Mental State Examination (MMSE) [31].

CI was determined using the MMSE questionnaire, abbreviated version, which includes 6 questions and with a maximum score of 19 points [31]. “Presence of CI” was determined in individuals with a score < 13 points [31]. CI tests were performed in a separate office in the morning to minimize external influences and were administered as an interview by trained nurses [32]. In the current study, CI was evaluated in terms of temporal and spatial orientation, short- and long-term memory, attention, concentration, abstraction, comprehension, memory and intelligence, executive capacity, and visuo-constructive capacity.

### Socio-demographic correlates

Sociodemographic data was collected from all participants, including sex (men and women), age reported continuously and analyzed also as categorical variable (60–69, 70–79, and > 79 years), education level (up to primary [< 8 years of studies], secondary [between 8 and 12 years of studies] and beyond secondary [> 12 years of study]), marital status (married and others that includes single, widowed or separated) and, indigenous ethnicity (yes and no) [11].

### Statistical analysis

Weights took into account the complex survey design, and the four levels of the multistage sampling. Descriptive data are presented as frequencies and means with standard deviation (SD) according to CI (no or yes). Chi-square tests (x^2^) for categorical variables, and student-tests for independent samples for continuous variables were used to compare the characteristics of participants according to the state of CI.

Multivariable logistic regression models were performed to estimate odds ratios (OR) and 95% confidence interval (95%CI) for the association between each independent variable (body mass index, metabolic syndrome, risk of cardiovascular disease, smoking, and physical activity) and CI (“yes” or “no”). Models were adjusted for the following potential confounders: region, geographical area, sex, age, education level, marital status, and indigenous ethnicity. Analyses were weighted by the survey design [11]. All statistical analyses were conducted using SPSS V22 software (SPSS Inc., IBM Corp., Armonk, New York, NY, USA). A significance level of p < 0.05 was adopted.

## Results

There were no significant differences (p > 0.05) between eliminated participants (n = 770) and participants of the current study by cardiovascular risk factors and CI. The total number of participants included in the study was 2031 (63.7% women) with a mean age of 71.0 (SD: 8.01) years. Overall, 47.8% of the participants were in the age group 60–69 years, 50.9% had studied up to primary school (≤ 8 years), 19.4% were married, 72.7% were classified as overweight/obese, 35.7% had metabolic syndrome, 33.6% had high risk of cardiovascular disease, 14.8% had never smoked, and 57.8% were physically inactive. There were significant differences (p < 0.05) between MMSE, age (continuous and categorical), education level, ethnicity, body mass index (continuous and categorical), risk of cardiovascular disease, and smoking by CI (Table 1).

**Table 1:** Characteristics socio-demographic by Cognitive Impairment in older adults

Variables	Cognitive Impairment		p-value
	No	Yes	
	n = 1784	n = 247	
MMSE, mean (SD)^1^	10.1 (2.5)	16.8 (1.9)	< 0.001
Age (years), mean (SD)^1^	70.3 (7.5)	76.1 (9.3)	< 0.001
Age categories, n (%)^2^			
60–69 years	926 (51.9)	72 (29.1)	< 0.001
70–79 years	610 (34.2)	82 (33.2)	
> 79 years	248 (13.9)	93 (37.7)	
Sex, n (%)^2^			
Men	639 (35.8)	98 (39.7)	0.237
Women	1145 (64.2)	149 (60.3)	
Education level, n (%)^2^			
Up to primary	859 (48.1)	175 (70.8)	< 0.001
Secondary	722 (40.5)	56 (22.7)	
Beyond secondary	203 (11.4)	16 (6.5)	
Marital status, n (%)^2^			
Married	666 (37.3)	118 (48.1)	0.746
Other	1118 (62.7)	128.9 (51.9)	
Indigenous ethnicity, n (%)^2^			
Yes	309 (17.3)	17 (6.6)	< 0.001
No	1475 (82.7)	230 (93.4)	
Body mass index, mean (SD)^1^	29.3 (5.3)	28.0 (5.2)	0.001
Body mass index category, n (%)^2^			
Underweight	191 (10.7)	52 (21.0)	
Normal weight	270 (16.8)	42 (17.0)	< 0.001
Overweight	615 (38.3)	78 (31.6)	
Obese	708 (44.2)	75 (30.4)	
Metabolic syndrome, n (%)^2^			
Yes	1122 (62.9)	156 (57.1)	0.209
No	662 (37.1)	118 (42.9)	
Risk of cardiovascular disease, n (%)^2^			
Low	379 (21.2)	34 (13.7)	< 0.001
Moderate	396 (22.2)	29 (11.5)	
High	1009 (56.6)	184 (74.8)	
Smoking, n (%)^2^			
Never	940 (52.7)	152 (61.5)	0.017
Ex-smoker	569 (31.9)	70 (28.3)	
Smoker	275 (15.4)	25 (10.2)	
Physical activity, n (%)^2^			
Inactive	949 (53.2)	154 (62.5)	0.058
Active	835 (46.8)	93 (37.5)	

Data presented as mean and SD for continuous variables or n (%) for categorical variables

^1^student t-test for independent samples; ^2^chi-square test

SD: standard deviation

**Fig. 2:**
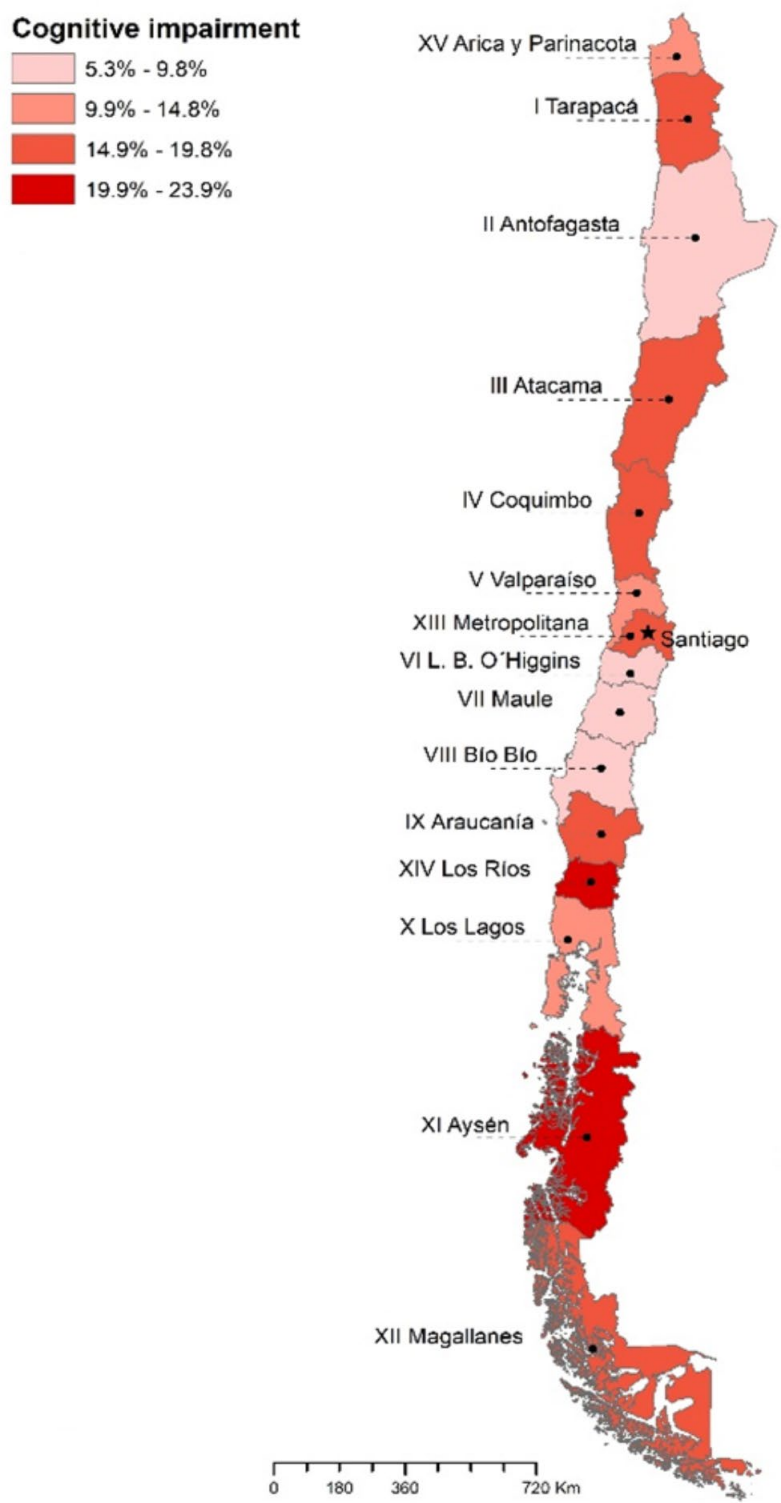
Geographic distribution of prevalence of Cognitive Impairment in older adults from Chile

Overall, the prevalence of CI was 12.2% at the national level. There were variations in CI prevalence across different regions, with some regions showing higher rates than others (Fig. 2).

**Fig. 3:**
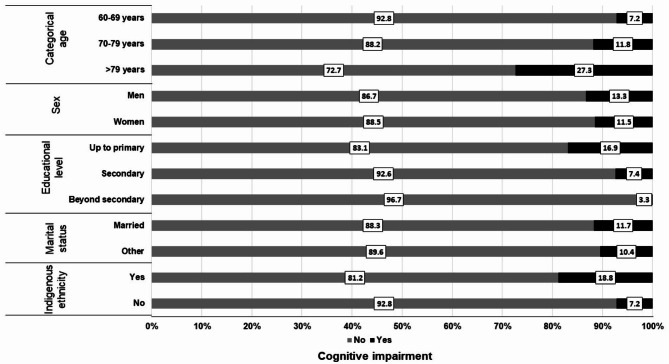
Proportion (%) of participants with Cognitive Impairment according to sociodemographic characteristics

**Fig. 4:**
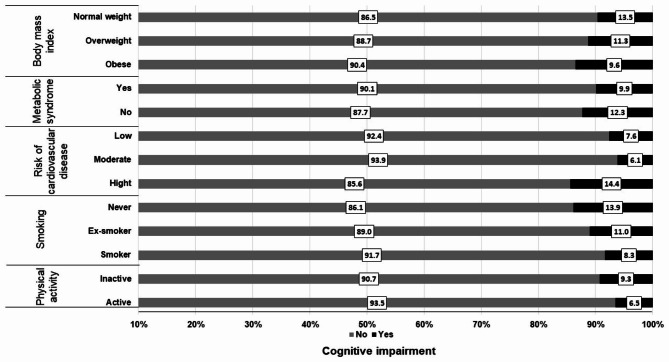
Proportion (%) of participants with objective cognitive function according to cardiovascular risk factors

Figures 3 and 4 show the proportion of participants with CI according to sociodemographic characteristics and cardiovascular risk factors. CI participants were likely to be older (> 79 years), men, participants with lower educational attainment, those married, and with indigenous ethnicity (Fig. 3). Furthermore, CI was more likely in participants with overweight and obesity, those without metabolic syndrome, with high risk of cardiovascular disease, never smokers and physically inactive (Fig. 4).

Associations between cardiovascular risk factors with and CI adjusted for region, geographical area, sex, age, education level, marital status and, ethnicity are shown in Table 2. High risk of cardiovascular disease was associated with higher odds of CI (OR: 2.04; 95%CI: 1.20–3.45) compared to low risk of cardiovascular disease. On the other hand, smoking was associated with a lower likelihood of CI (OR: 0.56; 95%CI: 0.36–0.87) compared to never smoking. Overweight and obese vs. normal weight, no vs. yes metabolic syndrome, and active vs. inactive were not associated with lower or higher odds of CI.

**Table 2:** Multivariable logistic regression for the association between cardiovascular risk factors with Cognitive Impairment in older adults

Variables	Cognitive Impairment		p-value
	OR (95%CI)		
Body mass index			
Normal weight	1.00		
Overweight	0.81 (0.54–1.21)		0.167
Obese	0.68 (0.45–1.01)		
Metabolic syndrome			
Yes	1.00		0.210
No	1.27 (0.87–1.84)		
Risk of cardiovascular disease			
Low	1.00		< 0.001*
Moderate	0.79 (0.39–1.62)		
High	2.04 (1.20–3.45)		
Smoking			
Never	1.00		0.018*
Ex-smoker	0.76 (0.56–1.02)		
Smoker	0.56 (0.36–0.87)		
Physical activity			
Inactive	1.00		0.059
Active	1.46 (0.98–2.18)		

Models were adjusted for region, geographical area, sex, age, education level, marital status, and indigenous ethnicity.

OR: odds ratios; CI: confidence intervals

*p < 0.05.

## Discussion

Using a representative sample of adults from Chile, our study examined the prevalence of CI in a nationally representative sample and verified the association of cardiovascular risk factors with CI in adults aged 60 years and older from Chile. In this sample 12% of participants had CI. Furthermore, we found that smoking was associated with a significantly lower probability of CI compared to never smoking, regardless of region, geographic area, gender, age, educational level, marital status, and ethnicity. Previous studies have consistently shown that current smokers have lower rates of CI compared to those who have never smoked (17.4% vs. 25.9%). However, in our study, we found that the prevalence rates of CI were even lower in current smokers (8.8%) compared to the group of ex-smokers (11.0%) and those who never smoked (14.4%) [33].

Cognitive dysfunction is defined as diminished or impaired mental and/or intellectual function [34]. Early identification of CI, through screening, would ideally allow patients and their families to receive care at an earlier stage in the disease process, potentially facilitating discussions regarding decision making while the patient still retains decision making capacity. Clinical experts and researchers have suggested that the health, psychological, and social benefits from early recognition of CI include: early education of patients and caregivers on the disease process; early coaching of caregivers in how to manage the patient; advanced planning; reduced patient and family anxiety and stress, as well as reduced caregiver burden, blame, and denial; patient safety; and promotion of advocacy for research and treatment development [35].

There is some evidence suggesting that cardiovascular risk factors are associated with several outcomes, including CI and mortality [36–39]. Few studies, however, have been conducted on the association between cardiovascular risks and CI among the elderly in Chile despite the fact that cardiovascular risk and CI in the elderly can have significant consequences for the country’s health system [40]. Chile presents a great regional variation in socio-economic development and disparities in health indicators [41]. Consequently, our study’s results show that the prevalence of CI and its increments are very heterogeneous within territories and even regions. The difference between subnational levels and smaller areas, represented by regions suggest geographic disparities in CI. Scrutinizing our results, health service in areas with higher CI prevalence coincide with less developed communes in Chile, according to the Community Development Indicator (CDI) [41], even in regions with better socioeconomic development. The above results reinforce the importance of examining prevalences at sub-national labels and the need to implement preventive measures at the local level to address the determinants of this problem.

The increasing prevalence of CI as an intermediate state between normal aging and dementia has important implications for public health planning, particularly in countries like Chile where the population is rapidly aging. As the number of older adult’s increases, the burden of CI on individuals, families, and healthcare systems is likely to grow. In overall, 12.6% of the adults from United States (≥ 44 years old) display decreased cognitive function, which is the beginning of the cognitive decline (Subjective Cognitive Decline Measures). In the current study, we found a prevalence of CI of 12.2% at the national level. However, our sample is for adults ≥ 60 years of age, which in Chile is considered older adults. In another study conducted in Spain, the estimated prevalence of adults ≥ 65 years of age with CI was 18.5%, showing values much higher than our study [42]. Comparing our findings from the 2016-17 National Health Survey with the previous National Health Survey conducted in 2009-10 in Chile, we verified that the prevalence of CI remained at 12.2% after almost 8 years [11]. Despite the consistent prevalence of CI between surveys, the elderly population has increased by 400,000 during the period, indicating a higher number of individuals with CI in absolute terms. Nevertheless, a study conducted by the Mayo Clinic Study of Aging, which followed individuals aged 70 and older for an average of 5 years, found that the rate of progression is in the range of 5–6% per year [43]. The increase in population age, therefore, may increase the prevalence of CI in the future. Epidemiological studies of CI aging in Latin America and in particular Chile, however, are still scarce compared to those of northern countries.

CI involves more than just the loss of memory, as it encompasses a range of cognitive functions. While some degree of memory decline is a normal process of aging, CI can lead to more severe conditions such as dementia and Alzheimer’s disease [44]. It is essential to recognize that CI affects multiple cognitive domains, including executive functions, visuospatial skills, and attention, making it a complex and multifaceted condition.

Proper assessment tools, such as the widely used MMSE, can help in detecting CI, although it’s important to acknowledge its limitations and consider more comprehensive assessments, such as the Montreal Cognitive Assessment (MoCA), which offers greater sensitivity and specificity in detecting MCI and other pathologies [45]. The causes are not clear; however, genetic, and environmental factors need to be considered. The main known risk factors that increase the chances of developing dementia or Alzheimer’s disease are family history, medical conditions, and lifestyle. There was no significant association between weight status and CI in the present study. In fact, the prevalence of CI was higher in normal weight participants (13.5%) compared to those who were overweight (11.3%) or obese (9.6%). A previous study in US Americans, on the other hand, found a relationship between BMI and poor cognitive functioning, specifically regarding attention, executive function, and general cognitive function. No associations, however, were shown for memory, which is considered a component of cognition to identify CI [46].

Scientific literature has already shown an association between obesity, inflammatory processes related to malnutrition (body mass index) and CI as well as mortality [47–49]. Additionally, low levels of physical activity have been positively associated with greater cognitive decline and mortality [50, 51]. Therefore, unintentional body weight gain in combination with physical inactivity may also be associated with CI function in elderly individuals [52, 53].

These results show significant associations between CI and risk of cardiovascular disease and tobacco use in older adults remain inconsistent [54–58]. Several studies have suggested that risk of cardiovascular disease increases or even aggravates the risks of a variety of mental health outcomes [6, 59, 60]. The mechanisms by which risk of cardiovascular disease may impair cognitive function in older adults, however, are not completely understood. Potential explanations include that long term cardiac diseases may alter the cardiac structure, causing, for example, left ventricular hypertrophy due to chronic hypertension, which is a potential mechanism for vascular dementia [60, 61]. This is one of many variables that could be altering vascular structures related to cognitive decline and dementia that occur particularly in aging [62].

Cardiovascular disease has also been linked to structural and functional changes in the brain and mind that may contribute to cognitive decline and dementia in aging, for example: The hippocampus is a key brain region involved in memory formation and consolidation. Studies have shown that cardiovascular diseases are associated with reduced hippocampal volume and decreased hippocampal function, which may contribute to objective cognitive function and dementia [63]. Further, cardiovascular diseases have been associated with changes in the white matter of the brain, which contains myelinated axons that facilitate communication between different brain regions. These changes can include a reduction in white matter volume, disrupted white matter tracts, and an increase in white matter lesions. Such alterations can contribute to cognitive decline and dementia [64, 65], indicating that cardiovascular disease have the potential to affect the integrity of the brain’s white matter and, consequently, its cognitive function.

Regarding other risk factors studied here, they were analysed as confounding variables in another study. The investigators demonstrated that the observed declines in cognitive function were not mediated by competing cardiovascular events, reinforcing their argument for an association between left ventricular hypertrophy and CI, independent of cardiovascular comorbidity and outcomes [66].

In this same sense, a study that followed more than 2600 elderly people who used the same instrument in our study to determine CI, shows that lifestyle can play an important role in the development of CI. Some modifiable characteristics and behaviors influence such as; no spouse, lower income, worse psychological well-being lower intake of fruits and fresh vegetables, more limitations in activities of daily living, and less commitment towards was significantly associated with CI [67]. Our data confirm that high cardiovascular risk is associated with a four-fold increase of the odds of having objective cognitive function impairment. However, it is important to note that smoking alone has not been proven to cause CI. A recent study suggests a higher risk of mortality due to cerebrovascular and respiratory diseases in older adults with mild CI who smoke [68]. Previous research has linked excessive cigarette use to psychological disorders, but a direct relationship with CI has not been established [69]. Furthermore, the literature has not been conclusive in this regard. While some detect higher rates of impairment in smokers, others detect the opposite or simply do not observe differences [70, 71]. Also, the lack of consistency in the studies is attributed to objective CI being a screening assessment, which results in multiple etiologies [9].

There were some limitations to this study: First, this study failed to establish causality. Second, this study did not evaluate the components of minimally, for example, neuropsychiatric problems, selective attention, processing speed and immediate and delayed memory, sensitive cardiovascular diseases. Third, although the MMSE questionnaire was validated in a Chilean sample and was used in this study with people over 60 years of age, the questionnaire was originally validated with a sample older than 64 years. Fourth, selection bias may be present due to the non-inclusion of elderly people living in non-institutional settings, nursing homes, or congregational care centres, which account for 17,000 elderly people throughout the country [72, 73]. Furthermore, one of the main limitations is related to the measurement of factors such as overweight and smoking. While data on overweight were directly collected, smoking habits were obtained through self-reported questionnaires, which may be prone to biases or measurement errors. As a result, the inconsistencies observed in these factors may be influenced by these limitations. Due to the cross-sectional nature of the study it is also not possible to establish causal relationships between the identified risk factors and CI. Additionally, the reliance on a specific assessment tool, such as the MMSE, may not fully capture the complexity of CI across various domains. Despite these limitations, we believe that our study contributes valuable insights to the field of CI research, and we hope that future studies will address these limitations to further advance our understanding of this important health issue.

The advantages of this study are that it used a representative sample of a national population and is one of the few national studies that verify the effect of the association value of CI and cardiovascular risk factors and cardiometabolic outcomes in a sample of Latin American adults. Future prospective analyses will be needed to assess a correlation between CI and cardiovascular risk factors using preclinical markers of the two pathophysiological conditions and future studies, including organisation of the complex physical illness and social needs, to support people affected by CI can have a huge effect when taken as a whole.

## Conclusions

Our findings highlight the importance of considering lifestyle habits when addressing cognitive function in older adults. We recommend that policy makers pay special attention to high cardiovascular risk, body mass index, and physical inactivity, and consider strategies to support the development of personalized interventions. These measures could have a significant influence on the cognitive health of Chile’s adult population.

## Data Availability

The datasets generated and analyzed during the current study are available in the database repository of the Epidemiology Department of the Chilean Ministry of Health. The repository can be accessed at: http://epi.minsal.cl/bases-de-datos/.
